# The role of long non-coding RNAs in nasopharyngeal carcinoma: As systemic review

**DOI:** 10.18632/oncotarget.14211

**Published:** 2016-12-26

**Authors:** Rongzhang He, Zheng Hu, Qingmei Wang, Weihao Luo, Jia Li, Lili Duan, Yuan-shan Zhu, Di-xian Luo

**Affiliations:** ^1^ Translational Medicine Institute, National & Local Joint Engineering Laboratory for High-through Molecular Diagnosis Technology, Collaborative Research Center for Post-doctoral Mobile Stations of Central South University, Affiliated the First People's Hospital of Chenzhou of University of South China, Chenzhou 432000, P.R. China; ^2^ Department of Clinical Pharmacology, Xiangya Hospital and Institute of Clinical Pharmacology, Central South University and Hunan Key Laboratory of Pharmacogenetics, Changsha, Hunan 410078, P.R. China; ^3^ Center for Clinical Pathology, Affiliated the First People's Hospital of Chenzhou, University of South China, Chenzhou 432000, P.R. China

**Keywords:** nasopharyngeal carcinoma (NPC), lncRNAs, tumorigenesis, therapeutic targets, biomarker

## Abstract

Recent development of cutting edge research found that long noncoding RNAs (lncRNAs) plays important roles in carcinogenesis and progression. In Southeast Asia and North Africa, nasopharyngeal carcinoma (NPC) is the most common aggressive squamous cell carcinoma. Nasopharyngeal carcinoma is most frequently occurring in males. However, nasopharyngeal carcinoma is caused by a combination of several factors as viral, environmental factors, and heredity. Till now, the potential pathway or mechanism of NPC is not well known. In our present review, we strongly emphasized on long noncoding RNAs (lncRNAs) and its significant role in nasopharyngeal carcinoma. It has been showed that lncRNAs regulate the development and progression of different types of cancers, including NPC. In addition, it has been found that chromatin organization, transcriptional and post-transcriptional events are regulated by lncRNAs. Our present review summarizes the roles of lncRNAs in nasopharyngeal carcinoma and provides an overview of the feasibility of lncRNAs as diagnosis, prognosis and potential treatment for NPC patients.

## INTRODUCTION

Nasopharyngeal carcinoma (NPC) is the most common squamous cell carcinoma involving epithelial lining of the nasopharynx. Nasopharyngeal carcinoma is predominant in North Africa and in South-east Asia, especially in China [1]. In addition, the incidence of NPC in western countries is very rare, almost less than 1/100,000. But in China, NPC is highly prevalent with an incidence of 20/100,000. In China, the new NPC case increases exponentially as sixty thousand new NPC cases were reported in 2015 [2]. Development and progression of NPC involves genetic susceptibility, environmental or stochastic factors and Epstein-Barr virus (EBV) infection. However, in nasopharyngeal carcinoma, EBV functions as a surrogate biomarker. Moreover, the recommended treatment for NPC is radiotherapy, in combination with chemotherapy [1]. However, after curative treatment by radiotherapy in combination with chemotherapy, no big improvement in the overall survival rate of NPC patients has been observed [3]. Therefore, in order to develop a curative treatment, it is most required to understand the molecular mechanisms underlying nasopharyngeal carcinoma [4]. Additionally, advancement of the high-throughput technologies allows us to concentrate on research of the lncRNAs.

LncRNAs are non-protein coding transcripts with >200 nucleotides and located in nuclear or cytosolic fractions. It has been showed that the quantity of lncRNAs and the quantity structural gene are same in human genome [5]. Several supporting evidences have shown that the dysregulated lncRNAs has a significant role in nasopharyngeal cancers [6–10].

In this review, we illustrate and discuss the recent advancement in research of lncRNAs and its significant role in NPC. The potential role of lncRNAs as biomarkers for NPC and diagnosis or therapeutic target for NPC treatment is discussed in detail.

## CLASSIFICATION OF LNCRNAS

**Figure 1:**
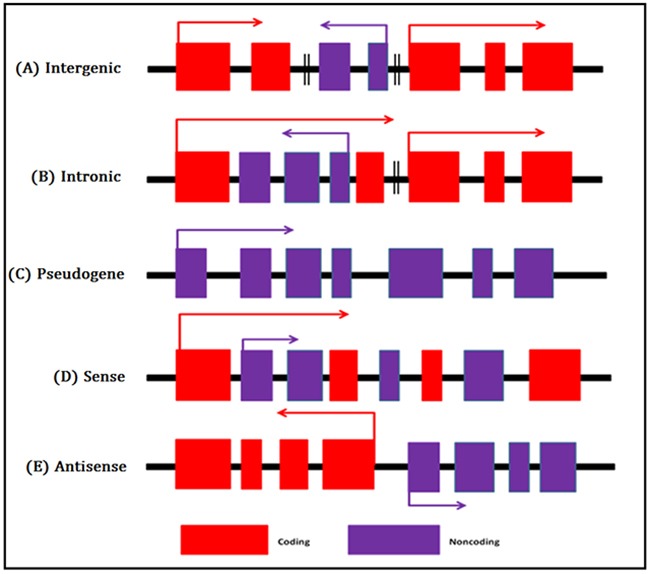
Overview of five broad categories of lncRNAs **A**. Intergenic lncRNAs are transcribed form intergenic regions. **B**. Intronic lncRNAs are transcribed from introns of coding genes. **C**. Pseudogenes are the “relics” of genes that have lost their coding function because of mutations. **D**. Sense lncRNAs are transcribed from the sense strand of coding genes and overlaps with a protein-coding gene. **E**. Antisense lncRNAs are transbribed from the antisense strand of protein-coding genes.

LncRNAs are non-protein coding transcripts with a length between 200bp to 10 kb [11]. On the basis of the GENCODE annotation, lncRNAs are categorized into five classes, as follows; sense, antisense, pseudogene, intronic, and intergenic lncRNAs [5, 12, 13] (Figure 1)

Moreover, nearly, 98–99% of human genome is non-protein coding regions. However, several mechanisms for transcription activation regulate the intergenic lncRNAs and intronic lncRNAs [12]. Sense lncRNA and antisense lncRNA are most frequent among all classes of lncRNAs [13].

## DYSREGULATION OF LNCRNA IN NPC

**Figure 2:**
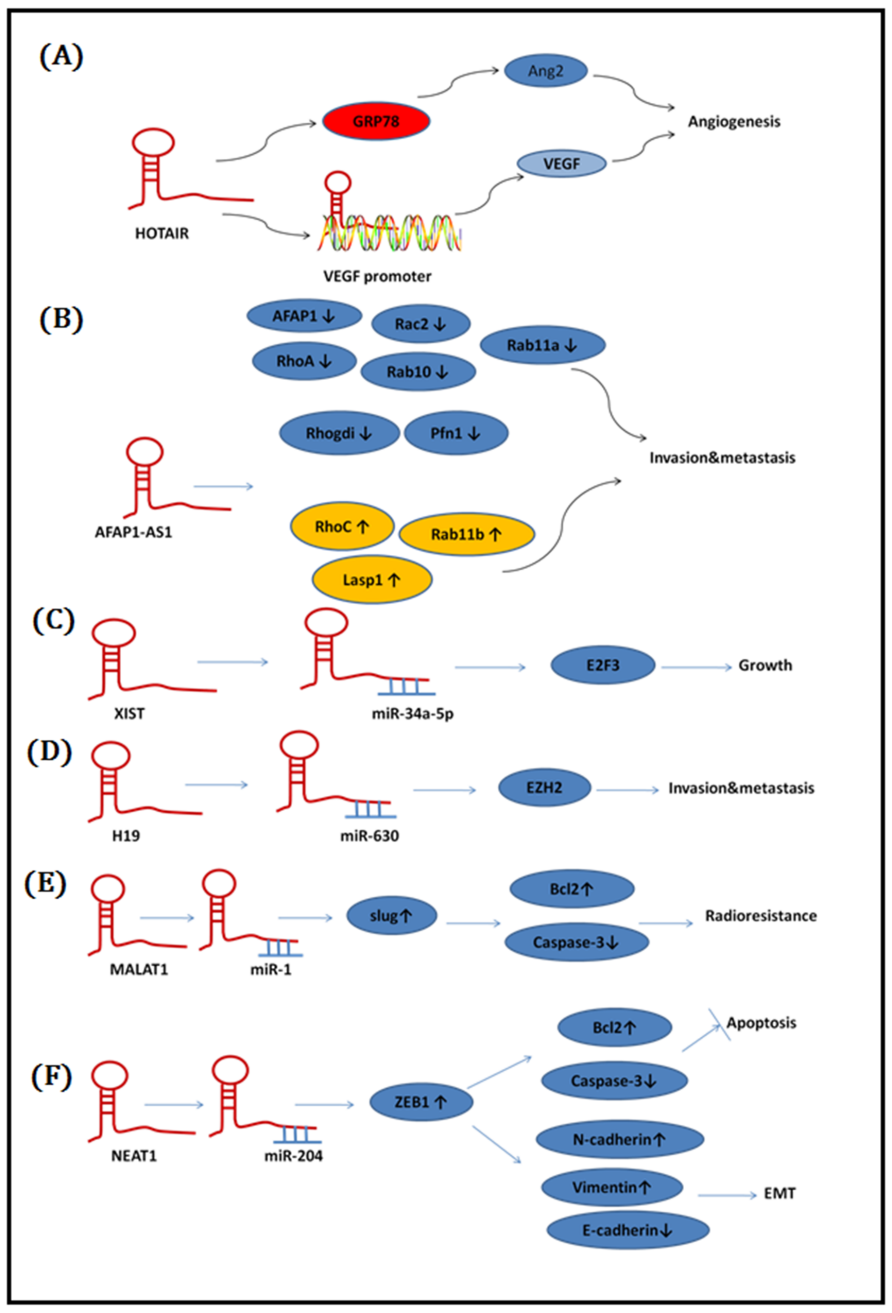
Functional mechanisms of lncRNAs in NPC **A**. Hotair promoted angiogenesis through directly activating the transcription of angiogenic factor VEGFA as well as through GRP78-mediated upregulation of VEGFA and Ang2 expression. **B**. AFAP1-AS1 inhibits AFAP1 protein expression and affected the expression of several small GTPase family members and molecules in the actin cytokeratin signaling pathway. promoted cancer cell metastasis via regulation of actin filament integrity. **C**. XIST functioned as an oncogene in NPC through up-regulating E2F3 in part through ‘spongeing’ miR-34a-5p. **D**. H19 Inhibits E-cadherin expression and promoted invasion via the mir-630/EZH2 pathway. **E**. MALAT1 regulates CSC activity and radioresistance by modulating mir-1/slug axis. **F**. NEAT1 regulates EMT phenotype and radioresistance by modulatin themir-204/ZEB1 axis.

It has been reported that lncRNAs is playing key regulatory roles in chromosome modification [14], transcription and posttranscriptional modification [15, 16]. We illustrate and provide an overview of the function of lncRNAs in the development of NPC (Figure 2). Here, we summarize all lncRNAs associated with NPC in Table 1.

**Table 1:** Roles and functions of lncRNAs in nasopharyngeal carcinoma

lncRNA	Location	Expression	Function in tumorigenesis	Classification	Potential mechanism	Reference
**XIST**	Xq13	Up	Oncogenic	Intergenic	XIST functioned as an oncogene in NPC through up-regulating E2F3 in part through ‘spongeing’ miR-34a-5p	[63]
**lncRNA-ROR**	18q21	Up	Oncogenic	Intergenic	Suppress p53 signal pathway and promotes proliferation, migration and chemoresistance	[69]
**AFAP1-AS1**	4p16.1	Up	Oncogene	Antisense	Inhibit AFAP1 protein expression and affected the expression of several small GTPase family members and molecules in the actin cytokeratin signaling pathway. promoted cancer cell metastasis via regulation of actin filament integrity	[9]
**Hotair**	12q13.13	Up	Oncogene	Antisense	Hotair promoted angiogenesis through directly activating the transcription of angiogenic factor VEGFA as well as through GRP78-mediated upregulation of VEGFA and Ang2 expression	[8]
**HNF1A-AS**	12q24.31	Up	Oncogene	Antisense	Promoted proliferation, migration and EMT	[37, 70]
**lncRNA-LET**	15q24.1	Down	Tumor suppressor	Intronic	inhibited NPC cells proliferation and induced cell apoptosis, transcriptional repressed by EZH2-mediated H3K27 histone methylation on the LET promoter	[71]
**H19**	11p15.5	Up	Oncogene	LincRNA	Inhibited E-cadherin expression and promoted invasion via the mir-630/EZH2 pathway	[54]
**NEAT1**	11q13	Up	Oncogene	Sense	Regulated EMT phenotype and radioresistance by modulatin themir-204/ZEB1 axis	[22]
**MALAT1**	11q13.1	Up	Oncogene	Sense	Regulated CSC activity and radioresistance by modulating mir-1/slug axis	[23]
**LOC401317**	NA	Up	Oncogene	NA	Inhibited cell proliferation and induced apoptosis	[10]
**LINC00312**	3p26	Down	Tumor suppressor	Intergenic	NA	[72]

### MALAT-1

It is a non-protein coding RNA with 8,000 nucleotide, located at chromosome 11q13. In addition, Ji et al first reported that expression of MALAT1 increased in no-small cell lung cancer (NSCLC) and associated with metastasis in NSCLC [17].

It was identified that MALAT1 is highly expressed in hepatocellular cancer [18], breast cancer [19], and colorectal cancer [20]. The radio-resistence is a major challenge for NPC radiotherapy. Previous studies showed that lncRNAs has important function in cancer radio-resistance [21, 22]. Recent study demonstrated that MALAT1 regulates radio-resistance [23] [24, 25]. It was demonstrated that MALAT1 directly bind to miR-1 in special site [23, 26]. Chou et al. found that MALAT1 regulate the metastases (migration and invasion) in breast cancer via affecting cdc42 through binding mir-1 [26], and which suggested that MALAT1 may regulate migration and invasion of nasopharyngeal carcinoma in similar pathways [27].

However, in renal cell carcinoma, high expression of MALAT-1 and the Livin protein were identified. Moreover, high expression of MALAT-1 leads to cell apoptosis and loss of the cell viability. MALAT-1 induced the high expression of the Livin protein which leads to proliferation and metastasis of renal cell carcinoma [17, 18].

### HOTAIR

Transcription of the antisense strand of HoxC gene leads to formation of HOTAIR, located on chromosome 12q13.13. HOTAIR was first reported as a polyadenyated and spliced transcript containing 2158 nucleotide with 6 exons [28]. Yan et al. reported that for nasopharyngeal carcinoma progression and survival, HOTAIR functions as an independent prognostic marker [28]. In previous study, it has showed that highly expressed HOTAIR is correlated with tumors size and clinical stage. In addition, the HOTAIR expression is exponentially increases with progression in clinical stage and finally high expression of HOTAIR is correlated with a poor prognosis of cancer. Moreover, the migration, invasion and proliferation of NPC cells is regulated by the expression of HOTAIR. Fu et al. found that HOTAIR mediated angiogenesis in NPC by direct and indirect signaling pathways [8]. It has been showed that, HOTAIR is up-regulated in NPC cells and tissues. Interestingly, HOTAIR knockdown causes inhibition of cell growth and angiogenesis. Additionally, HOTAIR, induced VEGFA by either binding to the promoter region of VEGFA or indirectly by GRP78-mediated up-regulation of VEGFA and Ang2 expression. Additonally, HOTAIR is a biomarker and therapeutic target for the treatment of NPC.

Presently, the functional mechanisms, regulatory network and pathways of action of HOTAIR remain mostly undiscovered. Recent study showed that HOTAIR interacts with polycomb repressive complex 2 (PRC2). In addition, HOTAIR also regulates chromosome occupancy by EZH2 (a subunit of PRC2). HOTAIR mediates trimethylation of the homeobox D locus by histone H3 lysine 27 (H3K27). The function of HOTAIR in regulation of chromosome occupancy which in turn enforces a silent chromatin state at Hox gene and other additional genes associated with cancer development and metastasis [28].

### AFAP1-AS1

However, AFAP1-AS1 was hypo-methylated and up-regulated in esophageal cancer by Wu and his colleagues in 2013 [29]. Later, several studies showed that AFAP1-AS1 was up-regulated and correlated with poor prognosis in different types of cancer [9, 30–36]. Bo et al., also identified that overexpression of AFAP1-AS1 was directly correlated with development of nasopharyngeal carcinoma [9]. AFAP1-AS1 knockdown causes inhibition of migration and invasive capability of NPC cell, while increased AFAP1 protein expression leads to loss of integrity for induced stress filament. Their data suggests that AFAP1-AS1 is a strong potential biomarker as well as a therapeutic target for the treatment of NPC.

Tumor formation ability of AFAP1-AS1 silenced SW480 cells was significantly suppressed. In addition, we found that AFAP1-AS1 silencing could promote the expression level of AFAP1 protein, a binding partner for oncogenic Src [29], while having no effect on the level of AFAP1 mRNA. AFAP1-AS1 knockdown could induce the loss of stress filament integrity, affecting the expression of Rho/Rac GTPase family members which finally correlated with the actin cytokeratin signaling pathway proteins in nasopharyngeal cancer cells [34–36].

### HNF1A-AS

HNF1A-AS, a lncRNAs, containing 2,455nucleotide with a single exon located at 12q24.31 [37]. It has been showed that HNF1A-AS played an important role in different types of cancers [37–40]. Recently, Zhuang et al. identified that HNF1A-AS is up-regulated and promoted cell proliferation and metastasis in NPC [37]. HNF1A-AS knockdown results in inhibition of cell proliferation both *in vivo* and *in vitro*. HNF1A-AS-knocdown induced cell get arrested in G0/G1 phase. Moreover, HNF1A-AS-knockdown leads to lower expression of EMT biomarkers. Therefore, it provides a new therapeutic strategy for NPC diagnosis and therapy.

### LncRNA-LET

LncRNA-LET is a transcript of 2606 bps length located on 15q24.1. It was reported that lncRNA-LET was down-regulated and acted as a tumor suppressor in several types of cancers [41–44]. Sun et al. reported that the level of lncRNA-LET was significantly decreased in NPC tissues. Moreover, down-regulated lncRNA-LET was directly correlated with size of the tumor, burden of the lymph node which in turn associated with clinical stages and poor survival. LncRNA-LET inhibited the proliferation of the NPC cells but induced cell apoptosis. Hence, lncRNA-LET is playing significant role in naso-pharyngeal carcinoma by proliferation of cells and apoptosis through an epigenetic mechanism.

In addition, recent study showed that lncRNAs-LET was functionally dysregulated in a variety of tumors. Moreover, the molecular mechanisms are still not clear. However, lncRNA-LET was repressed by hypoxia-induced histone deacetylase 3 through modulation of the lncRNA-LET promoter region [41, 42]. In addition, down-regulation of lncRNA-LET leads to stabilization of nuclear factor 90 protein which results into hypoxia-induced cancer cell invasion. Finally, unrevealing the function of lncRNA-LET allow us to open new avenues for therapeutic intervention against nasopharyngeal cancer [44].

### H19

H19 is one of the earliest-discovered and widely studied lncRNAs. Camilynn and his colleagues in 1990, has found that H19 is an imprinted gene [45]. However, H19 is up-regulated in patients with gastric cancer [46–48], bladder cancer [49], prostate cancer [50], colorectal cancer [51], glioma [52], breast cancer [53]. Recently, Li et al. identified that H19 was also up-regulated in patients with NPC [54]. It has been reported that H19 suppressed the expression of E-cadherin followed by invasion of NPC cells via the mir-630/EZH2 pathway. It has been showed that H19 plays a significant role in metastasis and a therapy target for NPC. However, in the future, more research is required to understand the correlation between the expression of H19 and the development of NPC.

### XIST

In addition, XIST is a spliced lncRNA transcript comprises of 19kb nucleotide, transcribed from *XIST* gene [55–57]. Recent studies have shown that XIST is required for the imprinted and random forms of X inactivation with gain or loss of function [58–60]. Since last several years, it has showed that XIST is found to be increased in non-small cell lung cancer [61], glioblastoma [62]. Recent report found that XIST was up-regulated in NPC tissues and acted as an oncogene [63]. Finally, XIST induced the expression of E2F3 through “spongening” mir-34-5p.

However, XIST interact with two nuclear proteins act as possible RNA chaperones [58, 62].

### Potential clinical applications of lncrnas in npc

According to the recent research, lncRNAs are considered as new type biomarkers of disease, as many lncRNAs showed species-specific and tissues-specific expression. In addition, lncRNAs are dysregulated in cancers and other diseases. Moreover, it is found that some lncRNAs are present in body fluid, and this suggests that circulation or secretory lncRNAs plays important roles in diagnosis as biomarkers. Merdan Fayda et al., found that plasma GAS5 could be a biomarker to predict the treatment response in HNC patients (including nasopharyngeal carcinoma) [64]. Moreover, exosomes are small membrane vesicles containing proteins, mRNA and miRNAs, and it suggests that they have a significant functions in cellular communication [65]. In addition, lncRNAs usually packed into cellular vesicles [66]. Furthermore, Gezer et al. have demonstrated that lncRNAs have a differential abundance in exsomes [67]. They found that several lncRNAs were accumulated in cellular vesicles in MCF7 and Hela cells.

LncRNAs may also be new targets for NPC therapy. As H19 is an oncogenic lncRNA and upregulated in multiple tumors including NPC, hence, it is a promising alternative therapy target of cancer [68]. H19 may also be a potential therapy target of NPC. Taken together, although only few lncRNAs have been characterized in NPC presently, and the dysregulated lncRNAs will be the most significant factor for the clinical diagnosis and follow-up treatment therapy of NPC.

### Summary and prospect

In this review, we briefly describe both previous and recent studies regarding the association of lncRNAs with NPC. However, expression of lncRNAs and their significant function in development of NPC are quite studied. LncRNA, also has an important function as novel biomarker for the clinical diagnosis and treatment of NPC. In future research, we must concentrate on the function of lncRNAs and their correlation with the development of NPC as well as development of new clinical diagnostic biomarker and therapeutic target of NPC. Most importantly, the clinical trials will be taken to study their effects in humans. In the future, lncRNAs will be the key factor in NPC biology with the effects.
